# Liking music with and without sadness: Testing the direct effect hypothesis of pleasurable negative emotion

**DOI:** 10.1371/journal.pone.0299115

**Published:** 2024-04-10

**Authors:** Emery Schubert

**Affiliations:** Empirical Musicology Laboratory, School of the Arts and Media, UNSW Australia, Sydney, NSW, Australia; Novi Sad School of Business, SERBIA

## Abstract

Negative emotion evoked in listeners of music can produce intense pleasure, but we do not fully understand why. The present study addressed the question by asking participants (n = 50) to self-select a piece of sadness-evoking music that was loved. The key part of the study asked participants to imagine that the felt sadness could be removed. Overall participants reported performing the task successfully. They also indicated that the removal of the sadness reduced their liking of the music, and 82% of participants reported that the evoked sadness also adds to the enjoyment of the music. The study provided evidence for a “Direct effect hypothesis”, which draws on the multicomponent model of emotion, where a component of the negative emotion is experienced as positive during music (and other aesthetic) experiences. Earlier evidence of a mediator, such as ‘being moved’, as the source of enjoyment was reinterpreted in light of the new findings. Instead, the present study applied a semantic overlap explanation, arguing that sadness primes emotions that share meaning with sadness, such as being-moved. The priming occurs if the overlap in meaning is sufficient. The degree of semantic overlap was defined empirically. The present study therefore suggests that mediator-based explanations need to be treated with caution both as a finding of the study, and because of analytic limitations in earlier research that are discussed in the paper.

## Introduction

A considerable portion of the population (estimates ranging from around 25% to 50%) will report that music they love can also make them feel negative emotions such as sadness [1–6]. This finding has mystified researchers. How can a loved activity simultaneously produce a negative feeling, and yet lead the same individual to eagerly seek out the experience?

### The Indirect effect hypothesis

Much theorising has been proposed to explain the conundrum as it applies to music listening and the contemplation of the arts in general. A dominating approach argues that the ‘sadness’ (the negative emotion that is the focus of the current investigation, and one that has received much attention) evoked by the music serves some non-negative purpose. The negative emotion is not in and of itself enjoyed. We will refer to such explanations as part of the ‘Indirect effect hypothesis’, meaning that a negative emotion such as sadness itself cannot or should not directly play a role in the generation of pleasure. The Indirect effect hypothesis is old, with written origins in Aristotle’s concept of catharsis from 4^th^ century BCE–where certain negative emotions in response to the arts act as a psychic cleanser, which removes bad or negative emotions from the soul [7,8]. The enduring concept of catharsis suggests an Indirect effect hypothesis because the negative emotion itself is not enjoyed directly. Rather, it is the cleansing, or the product of the cleansing that feels good. (Please note that in this article, the terms enjoyment, pleasure, feels-good, preferred, loved and liked are treated, more or less, as substitutable synonyms; see [9]) The negative impact of the emotion is thus compensated for by the positive effect on the soul or, in early 21^st^ century parlance, the mind.

A more recent version of the Indirect effect hypothesis is that sadness produces pleasure indirectly by triggering an intermediary step, sometimes referred to as a ‘mediator’. ‘Being moved’, for example, has been reported as the underlying reason for listening to otherwise sad music. Being moved can be seen as consisting of positive aspects, in addition to negative aspects [10–13]. It is the positive aspects of being moved that are responsible for the pleasure of the otherwise sadness-inducing music. Such explanations argue that the negative emotion occurs alongside a mediator, and so itself is not the direct cause of the positive aspects of the experience, thus eradicating the paradoxical aspect of the phenomenon.

A common technique to test the Indirect effect hypothesis is to ask participants to listen to a piece of music and rate the felt sadness and enjoyment experienced, in addition to rating the alleged mediator. If the enjoyment ratings are correlated with the mediator, and provided this correlation is overall stronger than is sadness with enjoyment, we have evidence, albeit correlational, that the mediator is the direct cause of the liking, not the sadness, supporting the Indirect effect hypothesis. To date, being moved has produced the strongest evidence of mediating sadness [3,14–16]. But other contenders that have been proposed, including beauty, wonder and nostalgia [for an overview, see 3, 17].

### Limitations of the Indirect effect hypothesis

An inherent weakness of Indirect effect hypothesis, and in particular the mediator-based explanation, is that it does not consider the phenomenal experience of the individual who claims that they both experience sadness, and that the sadness itself, for them, forms at least part of the pleasure [e.g., 6]. There are also limitations with research methods that are used to test the mediator explanation in the extant literature, as elucidated in the Method section.

Another limitation specifically concerns the mediator driven approach because it does not explain why the negative emotion would be present at all if it is the mediator that is driving the pleasure. If music is pleasurable because it is moving, and not because it evokes sadness, why would the listener not just seek the music that is moving but not sadness evoking? Is it because the mediator generates the negative (sad) emotion, as a by-product? But this would suggest that the occurrence of enjoyed negative emotion experiences such as sadness in response to music should be nothing more than an outlier, and be rarely reported as an enjoyed part of the experience (presumably well under the 25% of reports that are typical of published research, as indicated at the Introduction). Mediation theory therefore only explains why listeners claim to enjoy felt negative emotions to a limited extent. An alternative explanation is worth considering, and here the Direct effect hypothesis is proposed.

### The Direct effect hypothesis

The Direct effect hypothesis argues that there is something intrinsic about felt negative emotion evoked by music that attracts the listener, without mandating a mediator or some factor outside the negative emotion itself. The presence of accompanying affects (such as being moved) are not excluded, but they are not essential. One line of research that supports this hypothesis is the link between individual differences and enjoyment of sad music. Such research does not exclude the Indirect effect account, but it does suggest that individual factors attract the listener to sadness in music, raising the possibility that there is something peculiar about some negative emotions that allow them to be enjoyed in their own right.

Strong contenders for the disposition of people who enjoy the sadness evoked by music are empathisers, fantasisers, ruminators, those who demonstrate an openness to experience, and those with a high propensity to fall into states of absorption [2,3,16,18–22]. Current thinking is that these personal characteristics, especially empathising, absorption and openness to experience, allow the individual to connect with fictional narratives while suspending disbelief, and so exhibit a good capacity to “make-believe” [23,24], a capacity which generalises to emotions in music listening [e.g., see 16,25–27]. This explanation also presents an alternative theoretical perspective to the above cited literature, because rather than presenting sadness as a mere by-product of mediation or as a means to some beneficial end, the sadness can be ‘enjoyed’ for its own sake (directly). It is not real-sadness, but a make-believe, or aesthetic, kind of sadness, still experienced as sadness, but with some real-life negative aspect of the sadness not triggered [28].

The Direct effect hypothesis has a theoretical foundation. Emotion researchers such as Frijda [29] and Scherer [30] have conceptualised emotion as consisting of multiple phases or components operating in synchrony. This view is both reflective of contemporary understandings of emotion, and defined networks in the brain. In one instantiation of a componential model, Sander, Grandjean and Scherer [31] proposed five components/networks of emotion building on Scherer’s model: ‘Expression’ (e.g., a facial expression that communicates the emotion), ‘Action Tendency’ (e.g., motivation to approach toward, or flee from the cause of the emotion), ‘Autonomic Reaction’ (e.g., changed heart rate), ‘Feeling’ (what the emotion feels-like, such as ‘I feel sadness’) and ‘Elicitation’ (the internally triggered cause of the emotion through interpretation of environmental situation, association and instinct) such as prolonged loneliness eliciting sadness.

In the case of the enjoyment of negative emotions Schubert [32] proposed that when contemplating aesthetic stimuli the Action tendency component of an emotion is experienced as positive (motivation to approach) while other components remain as they would for real-life, non-aesthetic experiences of such emotions. The individual is not compelled to act in a withdrawn or aversive manner to the stimulus or event under contemplation because the perceiver has an implicit awareness that it is presented in an aesthetic or make-believe context. This dissociated response occurs because the individual has an intrinsic understanding of the safe, make-believe context in which the causal stimulus/event is perceived [33–35].

### Limitations of the direct effect hypothesis

The Direct effect hypothesis of enjoyment of negative emotion has arguably been difficult to test. If emotions happen to be correlated (such as sadness and being moved), researchers typically take this as an indication in favour of the Indirect effect hypothesis. But such interpretations do not exclude the possibility that the enjoyment directly stems from the sadness. While there is some evidence that those who enjoy negative emotion in music are indeed enjoying the negative emotion, there has been little systematic investigation of the experiential aspect of enjoyment of negative emotion in music. Other approaches to falsifying the Direct effect hypothesis are needed.

The approach taken in the present research is in the form of an ‘empirical thought experiment’, which has origins in so-called experimental philosophy [36]. Thought experiments, also referred to as mental simulation or ‘prefactual thinking’, rely on the participant’s capacity to imagine a situation and provide a response to that situation. The method can be particularly useful when a real-life stimulus-effect manipulation of interest is not possible or ethically compromising [e.g., 37]. It has been applied successfully to the empirical investigation of a range or research questions [38] and, of relevance here, to scenarios involving mental simulation of emotions [39–42].

Probing listeners to mentally simulate manipulating aspects of sadness induced by music is a simple approach to address both the Direct and Indirect effect hypotheses of enjoyment of experienced negative emotion in music. In brief, if a listener reports experiencing the sadness induced by a piece of music as pleasurable, the thought experiment to address the question of interest (to test if the sadness is the cause of the pleasure) is to ask the participant to imagine that the felt sadness, and only the felt sadness, can somehow be removed. If enjoyment is consequently diminished (as a result of the mentally simulated, excised sadness), the Direct effect hypothesis will be supported. Assurances would need to be set in place that the sadness was experienced (felt) and not just expressed by the music [43], and that the music was responsible for triggering the sadness, not some (extramusical) association (as discussed in the Method section).

## Aims

The aim of this study was to investigate whether negative emotion in music, in this case sadness, can be both experienced and enjoyed. Two competing hypotheses were tested:

H1 –the Indirect effect hypothesis, which predicts that: Sadness *removed* from a liked piece of music will *increase or not change* enjoyment. This is because it is not the sadness that is enjoyed, but something external to the sadness, such as being moved or some other mediator.

H2 –the Direct effect hypothesis predicts that: Sadness *removed* from a liked piece of sadness will *decrease* enjoyment. This is because the sadness itself is somehow enjoyed, regardless of the impact of correlated variables (such as being moved, etc.).

## Method

### Methodological and data analysis issues

This preamble to the method examines four key issues encountered in extant methods and data-analysis conventions stemming from controversy about use of experimenter- versus participant-selected stimuli. These issues are: Confounding extramusical association, Phenomenon of interest, Demand characteristics and Prospective mediators. This is followed by a discussion of problems that have emerged in experimenter-selected stimulus, and, as a result, a justification for the use of participant-selected music is then presented.

#### Confounding extramusical association

There has been growing consensus that investigations of enjoyed sadness in music should be assessed through experimenter-selected music. Participant- or ‘self’-selected music has the disadvantage that the music can have personal or other non-musical associations, meaning that it is not the music that is directly responsible for triggering sadness, but previously formed, ‘extramusical’ associations with the music. Self-selected music could therefore lead to *confounding extramusical associations* that evoke sadness: the music acting as a mere go-between with the external cause of the sadness and the experience of sadness, and therefore potentially lead to false conclusion of negative emotion being caused by the music. Furthermore, self-selected music does not assure that findings would be generalisable to other participants who did not self-select the same piece. Self-selected music is inevitably music that is familiar. Personal meanings and associations with familiar music could well lead to idiosyncratic responses, peculiar to one or a small number of individuals [for a detailed discussion on limitations in use of familiar music, see 44].

Although one of the main drivers for using experimenter-selected music is to avoid *confounding extramusical associations*, it is possible that even for unfamiliar (experimenter-selected) music a participant will have an emotional response to music because it triggers an external factor, rather than emanating from the music itself [45]. For example, while Day and Thompson [46] found that familiar music is more successful at evoking visual imagery (and hence increasing the likelihood of extramusical emotional associations), they also observed the important role of fluency, where music that is complex (low in fluency) is more likely to trigger visual imagery than music that is less complex (high in fluency), regardless of familiarity. Furthermore, autobiographical memories have been reported to be triggered by unfamiliar music, although to a lesser extent than familiar music [47,48, see also 49]. Thus experimenter-selected music can help to diminish the likelihood of data pollution through *confounding extramusical associations*, even if not eliminate it.

#### Phenomenon of interest

Use of unfamiliar music that is rated by an independent panel, or some other means, as evoking sadness and being pleasurable has been proposed to remedy the problem of *confounding extramusical association* [e.g., 14,16]. However, this approach also has its shortcomings. Others deciding what music is likely to evoke sadness will not necessarily evoke sadness to a sufficient degree in a randomly sampled participant to address the *phenomenon of interest* (enjoyment of evoked negative emotion in music). It is well documented that familiar music can evoke stronger emotions than unfamiliar music, with self-selected music being a particularly effective way to elicit the strong emotions [e.g., 43,50–56]. Similarly, others deciding what music someone likes is riddled with problems. Music preference calls into play several factors such as familiarity [57], making the assumption of an absolute, objective rating of pleasure in response to a given piece of music problematic. This constitutes a considerable drawback of experimenter-selected design because additional precautions need to be taken to assure that participant experiences capture the *phenomenon of interest* (both strong liking and experiencing of sadness), as discussed below.

#### Demand characteristics

Another problem with self-selected music is that it may attract *demand characteristics* bias. This bias can occur when the participant infers the research question [58,59]. For self-selected music the research objective can be inferred by the participant, in particular if they are asked to select music that they love that also evokes sadness. In this situation, the participant may guess that the study is concerned with enjoyment and experiencing sadness. If consciously or subconsciously they wish to please the experimenter, they may inflate their assessment of the amount of enjoyment the music generates or the amount of sadness it evokes or both. Furthermore, during participant recruiting, if mention is made that people are sought who experience sadness in response to loved music, it is self-evident that the participant pool will be biased, because only those who have the targeted experience are likely to participate, overlooking the opportunity to estimate how common the phenomenon is in a general population.

#### Prospective mediators

Overall, the studies adopting experimenter-selected designs have used interval rating scale measurements of the variables of interest (enjoyment, sadness, and the prospective mediator variables, such as being moved). In addition, other variables are rated to help reduce the likelihood that the participant will successfully intuit the aim of the study, and to capture information about alternative, prospective mediators. Interval rating scales have the advantage of being convenient for correlation based data processing procedures, such as statistical mediation analysis [60].

#### Problems with experiment-selected designs

Although research using experimenter-selected music designs have claimed to manage several methodological problems identified in self-selected music designs to address the current research question, as summarised above, experimenter-selected stimuli based approaches nevertheless have their own limitations (some overlapping with self-selected music approaches).

As mentioned above, experimenter-selected music is less likely to evoke strong emotions compared with self-selected music, and so it is possible that a person who is capable of experiencing intense sadness in response to loved music will not have that experience for music selected by the best-intentioned experimenter. Even with self-selected music, some studies have shown that only about one quarter to one third of participants report experiencing negative emotions such as sadness in response to music they love (see Introduction). Schubert (6) used the self-selection approach while considerably circumventing the problem of demand characteristics. He asked participants to select a piece of music that they love, but not revealing the research interest in negative emotions. As it turned out, about one third (25/73) of the participants spontaneously reported experiencing negative emotions, with specific mention made of sadness in 12/72 (i.e., one sixth of) cases (p. 17). In that study it was not clear, however, whether the sadness emanated from the music itself, or through some *confounding extramusical association*. Nevertheless the method mitigated *demand characteristics* bias, and above all, it ensured that the piece selected was highly liked, something which experimenter-selected approaches rarely guarantee. Konečni [61] also argued that fully-fledged aesthetic experiences in response to music are rare even under regular listening circumstances. Therefore, the *phenomenon of interest* would occur in an even smaller proportion of cases in studies applying experimenter-selected music, even if the stimuli have been previously screened for sadness evocation and enjoyment by individuals other than the participant them/her/himself.

Another related limitation of studies using experimenter-selected pieces concerns the response format itself, which commonly employs an integer-based rating scale for each of the affective variables of interest. The problem is not the use of rating scales *per se*, but the tradition of publishing rating scale results. Studies typically report scale (i.e., item) mean (X) and standard deviation (SD) scores, and/or the correlation coefficient (usually the Pearson product moment coefficient, r) for pairs of variables. The chief problem with such reporting is they imply assumptions about the distribution of the responses. Providing these descriptive statistics, and in particular when the data are then applied to parametric statistical analysis procedures, infers that the distribution of the data are normal, have homogenous variance and are linear [62, p. 311]. If these assumptions are taken at face value, it means that the density of responses diminish as data points are located further away from the mean, with the diminution per scale step being more rapid when the standard deviation is small. Consequently, when there is no explicit information provided about the nature of the distribution, the number of responses that meet the criterion for the *phenomenon of interest* could be relatively small, and risk not providing statistically sufficient power for meaningful analysis. A simple visual diagnosis can be made through scatterplots of felt sadness versus liking ratings. The decision needs to be made as to where the cut off mark is for sadness and liking scores above which count as satisfying the *phenomenon of interest*.

This weakness in extant research constitutes the most serious problem of the mediation-based explanation, which, to the author’s knowledge, has exclusively employed experimenter-selected stimuli and use of interval rating scales with X/SD/r reporting, assuming that any amount of sadness evoked by a piece of music should be proportionally implicated in its enjoyment. The assumption is incorrect because it asserts that a linear relationship is evidence of the *phenomenon of interest*. In fact, the *phenomenon of interest* is not concerned with enjoyed that accompanies low levels of sadness because when sadness levels are low, other reasons for enjoying the music are still perfectly viable. Evidence of this problem is reflected to some extent by the generally low correlations reported between sadness and liking scores, usually with a small effect size [r < .3, see 63]. When the correlation coefficient is small, no conclusion can be drawn about the *phenomenon of interest* because low correlation only reveals a lack of (non-zero) linearity, rather than information about the modality of the bivariate distribution. That is, a small correlation coefficient provides no information regarding the location of the mode of the distribution, or whether a desirable mode (also) exists in the high sadness, high liking region of the distribution.

In short, by not diagnosing the nature of the bivariate response distribution, the analytic approaches adopted for currently available experimenter-selected designs potentially exclude cases of high evoked sadness that accompany high liking, meaning that they have not captured the *phenomenon of interest* and so cannot make conclusions about it, or should do so with caution. One solution for future research employing ratings for all variables of interest while maintaining the advantages of the experimenter-selected stimuli approach is to recruit a sufficiently large random sample so that enough cases happen to fall in the desired range spontaneously. However, using self-selected music is more efficient because the *phenomenon of interest* is achieved by categorical self-selection.

#### Using self-selected stimuli–justification

With the above arguments, the stimulus self-selection approach can be justified provided some modifications are made to the way the approach has been applied in the past. These are itemised here in six points. Based on the above overview, the main innovations to note are points 2, 3c, 3d and 4. Square bracketed text following each point indicates the main methodological issue(s) discussed above that are addressed by each of the proposed actions.

1. Correspondence used for recruiting participants is not to indicate that the study is concerned with experiencing sadness in music, its enjoyment, or both [as per recommendations by 58, 59]. [Demand characteristics]
2. During the study, request that the participant selects music that is loved, not just liked, to ensure that the desired (high) liking category of music is attained [64]. [Phenomenon of interest]
3. Each self-selected loved, sadness-evoking piece is carefully screened to ensure:
  - that the music is highly liked,
  - the sadness is indeed felt,
  - the sadness emanates directly from the music, and not through extramusical association, and
  - the experienced sadness is implicated in the enjoyment of the music. [Confounding extramusical association; Phenomenon of interest]
4. A control condition is employed, for example where instead of requesting sadness-evoking music, music evoking another emotion that is not paradoxical is requested, such as a mediator proposed in previous research. An obvious choice is moving music (that is loved). [Demand characteristics; Phenomenon of interest]
5. A number of affect terms, including sadness and the control condition emotion should be added to a list of emotions rated in both test and control conditions to allow for comparison, and help identify prospective mediators. [Prospective mediators]
6. Since participants are explicitly asked to have potentially powerfully sad emotions evoked, towards the end of the study an additional stimulus is rated that requires evocation of a positive emotion. This satisfies potential ethical concerns where sadness experience could influence mood negatively, and allows the option of further comparisons with affects in the test condition that were prospective mediators. [Prospective mediators]

### Participants

103 participants, recruited from an English speaking tertiary institution, consisting mostly of undergraduate music students, completed the study. They were randomly assigned to one of the two conditions. Fifty participants were randomly assigned to the Sadness condition and 53 to the Moving condition in a between-subjects design. The research received ethics approval from the UNSW Australia institutional review board Human Research Advisory Panel B: Arts, Architecture, Design and Law. Participants were recruited from June 4, 2021 until June 9, 2021. Consent to participate was provided at the opening of the online survey, with a checkbox selected if the participant agreed to participate. No minors participated in the study.

### Materials

The Qualtrics survey platform (https://www.qualtrics.com) was used for human data collection. Self-selected music was identified through online links searched for and reported within the survey by the participant. The participant used an electronic device, such as a laptop, iPad or tablet. They were encouraged to wear earphones to listen to music, but this was not enforced. Affect terms consisted of a list of terms that are drawn from Schindler, Hosoya [65] and Schubert [66], as presented in the Procedures.

### Procedure

Prior to commencing the study, informed consent was requested verbally through the online interface, with all participants being asked to read an online participant information sheet, which included information about being free to withdraw from the study at any time. They were informed that their data would be treated confidentially, and were encouraged to ask questions if needed, and then to indicate if they wished to commence the study. Participants were randomly assigned into a Sadness (test) or Moving (control) condition. We describe the sadness condition here, but the moving condition is identical, except that ‘sad’ and ‘sadness’ is replaced with ‘moved’/’being moved’ and ‘movingness’ (respectively). Otherwise, where grammatically straight-forward ‘[CONDITION]’ is shown, which was replaced by ‘sadness’ or ‘moved’/’being moved’, depending on the assigned condition. After the tasks for the test or control condition were completed, all participants were invited to select another piece, but this time one that made them feel happy. Although this step of the study was completed by all participants, it will be referred to as the Happy ‘condition’ for convenience. The steps of the study are listed below. They followed one another in sequence, and the participant could not return to a step once they had answered the questions in that step and progressed.

- Participants were asked to **self-select a piece** that they both loved and that evoked sadness. They were encouraged to think about this for a few minutes if necessary. For those who could not come up with a piece that met these criteria, some alternative pieces were proposed, from which they could select, or, have further opportunity to select another piece. **Details** of the piece were collected.
- **Enjoyment** of the piece was rated: "How much do you like this piece?” (anchors: 0 = dislike it a lot; 100 = like it a lot)
- **Open-ended felt emotions** requested: “Please indicate in as much detail as possible any emotions that you feel in response to this piece. Be sure to include [CONDITION], of course.” (Free text response.)
- **Affects felt**. 26 felt affect terms were rated on a 3-point scale (A lot, A little, Not felt) on the extent to which each terms was felt. The wording of each terms was presented to the participant as—1: Being absorbed/completely immersed in the music; 2. Anger; 3. A sense of awe; 4. Feeling of beauty; 5. Calm; 6. Chills; 7. Compassion; 8. Empathy; 9. Euphoria; 10. Fear; 11. A feeling that is sublime; 12. Goosebumps; 13. Grief; 14. Happiness; 15. Joy; 16. Being moved; 17. Nostalgia; 18. Peacefulness; 19. Powerful feelings; 20. Release or relief (sometimes referred to as ’Catharsis’); 21. Sadness; 22. Tears/wanting to cry/feeling like crying/actually crying; 23. Tenderness; 24. Transcendence; 25. Tragedy; 26. Wonder.
- **Confirm felt and direct**. Confirm that: Affect terms marked as present in the previous step (‘A lot’ or ‘A little’) were (a) *felt* and (b) that they were triggered *directly* by the music, not by thoughts, memories, images, etc. (Yes/No for each of (a) and (b)).
- **Sadness removed**. Participant was instructed “Imagine that the felt sadness, and ONLY the felt sadness directly triggered by the piece could somehow be removed, with all your other felt emotions remaining intact. If this removal were possible, how do you think it might influence your liking of the piece?” There were 5 response options: I would like the piece a LOT LESS;
  I would like the piece a LITTLE LESS;
  It would make NO DIFFERENCE;
  I would like the piece a LITTLE MORE;
  I would like the piece a LOT MORE.
- **Affects that add to liking**. The same 26 Affect terms listed in step iv were rated on a 3-point scale (Adds to the pleasure, Does not add to the pleasure, Don’t know/not relevant) to assess whether the “the felt emotions add to the liking, pleasure, attraction or enjoyment”.
- **Cooling down.** The above procedure was repeated for a self-selected happy piece, but without any ratings of the 26 Affect terms requested (i.e. steps iv, v & vii excluded).
- **Background** (age, gender, music background) data were collected after which the participant was thanked and farewelled.

Some researchers, such as [67,68], treat the concepts of affect and emotion as distinct. In the present study the distinction is partly made for the convenience of distinguishing between participant open-ended response in step iii (emotion) versus their selection from a predetermined list of terms in steps iv, v & vii (affect). The term ‘emotion’ rather than ‘affect’ was used in all of these instruction steps because the former term was considered better understood by participants, regardless of whether referred to as emotion or affect in this article.

## Results

### Data validation

#### Participant profile by condition

Inferential tests demonstrated that the Sadness and Moving groups were statistically identical in terms of gender, age and years of music lessons (Table 1). Also comparable across the groups was the overall rating of liking, averaging over 90 on a 0–100 scale, with upper quartiles (Q3) demonstrating a ceiling effect in both conditions which supports the use of self-selected music for generating high levels of pleasure.

**Table 1:** Comparisons of sadness and moving condition participant characteristics.

Variable	Sad Condition (SD)	Moved Condition (SD)	Inferential test statistic (dof)	p	Effect size
N	50	53			
Gender			χ2(3) = 3.792	.285	w = 0.1919
Female	33	30			
Male	8	16			
Non-binary	1	0			
Not-reported	8	7			
Age (years)	23.76 (8.516)	22.86 (6.940)	F(1,86) = 1.351	.248	η^2^ = 0.015
Music lessons (years)	5.55 (6.014)	6.80 (5.365)	F(1,86) = 1.069	.304	η^2^ = 0.012
How much do you like this piece? (0–100)	90.08 (11.622)	93.21 (8.242)	t(99) = 1.568	.120	Cohen’s d = 0.312
Liking rating quartiles Min value:Q1:**Q2(median)**:Q3:Max value	69:90:**95**:100:100	51:82:**93**:100:100			
Confirmation that emotions were felt (Yes/No answer alternative)	n [yes] = 48 [96.2%]	n [yes] = 51 [96.0%]			
Emotions present triggered directly by the music (Yes/No answer alternative)	n [yes] = 45 [90.6%]	n [yes] = 48 [90%]			

#### Check that the emotion was felt and evoked emotion was directly due to the music

There was overall high confirmation that the emotions were felt (over 96% of participants) and over 90% of participants in both conditions confirmed that the sadness was triggered intrinsically by the music (not triggered by something outside the music). See Table 1 for breakdown by condition. Overall, participants from both conditions were successful at experiencing the target emotion (Sadness or Being moved) and confirmed that, as requested, the music was directly responsible for triggering the emotion, rather than due to some extramusical factor. All participants were retained for further analysis.

#### Most frequently reported music excerpts

All participants selected a piece that met the music selection criteria. Although researcher-suggested pieces were prepared in case a participant could not identify a self-selected piece meeting the criteria, none of the participants requested the researcher-suggested option, and so the research-suggested options were never used in the study. A selection of the self-selected items is presented in Table 2, showing composers/artists reported by at least three participants across the cohort, and listing the works reported at least twice across the cohort. Interesting similarities can be observed across conditions, with composers Beethoven, Chopin and Debussy, and artists Taylor Swift and Bon Iver appearing in the Moving and Sad conditions. Furthermore, for the Beethoven, two pieces were mentioned in both of these conditions: *Für Elise* and *Moonlight Sonata* (1st Movement). These selections reflect the shared tastes across the groups, and at the high proportion of musicians, in particular pianists, who participated (all of the more frequently selected Beethoven, Chopin and Debussy pieces were for piano). Table 1 reveals the overall high average years of music lessons reported across the cohort [69]. These selections also indicate the capacity for the same piece of music to evoke different emotions (being moving and sadness).

**Table 2:** Most frequently reported composers/artists and pieces by condition.

Moving condition	#	Sad condition	#	Happy condition	#
• Ludwig van Beethoven	3	•Ludwig van Beethoven	5	• Binki	3
- Bagatelle No. 25 in A minor, *WoO 59* Für Elise	1 1	- Bagatelle No. 25 in A minor, *WoO 59* Für Elise	2	- Sea sick	3
- *Sonata*, *Op*. *27*, *No*. *2*, ‘*Moonlight’*, i. Adagio sostenuto Movement	1 1	- *Sonata*, *Op*. *27*, *No*. *2*, ‘*Moonlight’*, i. Adagio sostenuto Movement	2	• Kero Kero Bonito - Trampoline	3
• Frederick Chopin	5	• Frederick Chopin	3	• Wolfgang Amadeus Mozart	5
- *Ballade No*. *1 in G Minor*, *Op*. *23*	4			- *Marriage of Figaro*, *K*. *492*	2
• Claude Debussy *- Suite bergamasque L*. *75*, Clair de lune	2 1	• Claude Debussy *- Suite bergamasque L*. *75*, Clair de lune	3 1	- *Piano Sonata No*. *11 in A major*, *K*. *331*, iii. Rondo alla Turka	2
• Bon Iver - Calgary	2 2	• Bon Iver	1	• Taylor Swift - Love Story	3 2
		• Taylor Swift	3	• Antonio Vivaldi	3
				- *Concerto No*. *1 in E major*, *RV 269*, ‘*Spring’*, i. Allegro	3

Note

Composers/artists reported at least three times are shown. Pieces reported at least twice are shown. # denotes number of participants who made the selection. Entries per column are in alphabetical order by artist/composer surname or last name.

### Emotion profile of sad music: Open-ended

After selection of a piece in their assigned condition, participants were asked to provide free descriptions of the emotions they felt in response to the selected piece (self-selected sad or self-selected moving music). The reported terms were pre-processed by identifying all reported emotion terms (participants could report more than one), correcting spelling mistakes, checking context and lemmatizing terms. This was followed by a frequency count of these terms for each condition. The target emotion was expected to be reported frequently in each condition.

Table 3 lists the emotion terms in descending order of frequency for each condition (including the Happy condition, where the same task was requested of participants in both conditions, but for a happy piece), with the most frequent words shown (down to a count of five). The selection of most frequent terms shown with an asterisk in the top rows of the table (above the horizontal cell divider) was determined by the ‘Power Fitted Elbow’ (PFE) technique that builds on word frequency distribution characteristics [70–73]. The expected target emotion (shown in italics font in the table) is reported most frequently in all conditions. Noteworthy is that sad was reported frequently in the Moved condition, while negative emotions were reported exclusively among the most frequently reported Sad condition emotions. Nostalgia was frequently reported in all conditions. In the Sad condition, the lemma Moved (not shown in the table) was mentioned 4 times, but was not reported frequently, according to the PFE criterion. Another interesting finding is that none of the frequently investigated mediator emotions (Being moved, in particular), appear in the most frequently reported items of the Sad condition list (sad, nostalgia, loss, melancholy and lonely). In contrast, the Moved condition did lead to frequent open-ended reporting of sadness.

**Table 3:** Most frequently reported open-ended emotion words reported in each condition.

Moved Condition	Count	Sad Condition	Count	Happy Condition	Count
*moved* *	21	*sad* *	43	*happy* *	70
happy*	13	nostalgia*	12	joy*	25
sad*	13	loss*	10	excitement*	21
nostalgia*	11	melancholy*	10	energetic*	19
joy*	11	lonely*	8	nostalgia*	10
excitement	10	hope	7	calm	7
calm	9	excitement	5	dance	7
hope	9	heart	5	love	7
energetic	8			awe	5
serene	7			cool	5
peace	6			fun	5
awe	6			optimism	5
beautiful	5				
anticipating	5				
comfortable	5				

Note

Lemmatised words are listed in descending order of frequency, and in alphabetical order within each frequency count. Only lemmas with counts of 5 or greater are displayed.

* Emotion words reported a reliable number of times according to the Power Fitted Elbow criterion.

Italics font denotes the term which best corresponds to the target emotion.

Like-colour cells are used to aid identification of frequently reported emotion words in more than one of the conditions.

### Emotion profile of sad music: Felt Affect term ratings

After open-ended responses were reported, participants were asked to indicate the extent to which each of 26 affect terms were felt when listening to the music. Again, the target affect terms were expected to be rated highest. The ratings for each affect term within and between conditions were examined.

A mixed ANOVA was conducted using rating as the dependent variable, with two independent variables: affect term (within-subjects factor, 26 levels), and condition (between-subjects factor, 2 levels). There was no significant main effect by condition (F(1, 99) = 6.706, p = .115, $\eta_{P}^{2}$ = 0.025). There was a main effect of affect term (F(25, 2475) = 32.135, p < .001, $\eta_{P}^{2}$ = .245). The interaction was also significant (F(25, 2475) = 7.191, p < .001, $\eta_{P}^{2}$ = .068). These findings indicate that heterogenous pairs of affect terms differed, both within condition and between conditions. Of particular interest are the differences between Affect terms within each condition.

**Fig 1:**
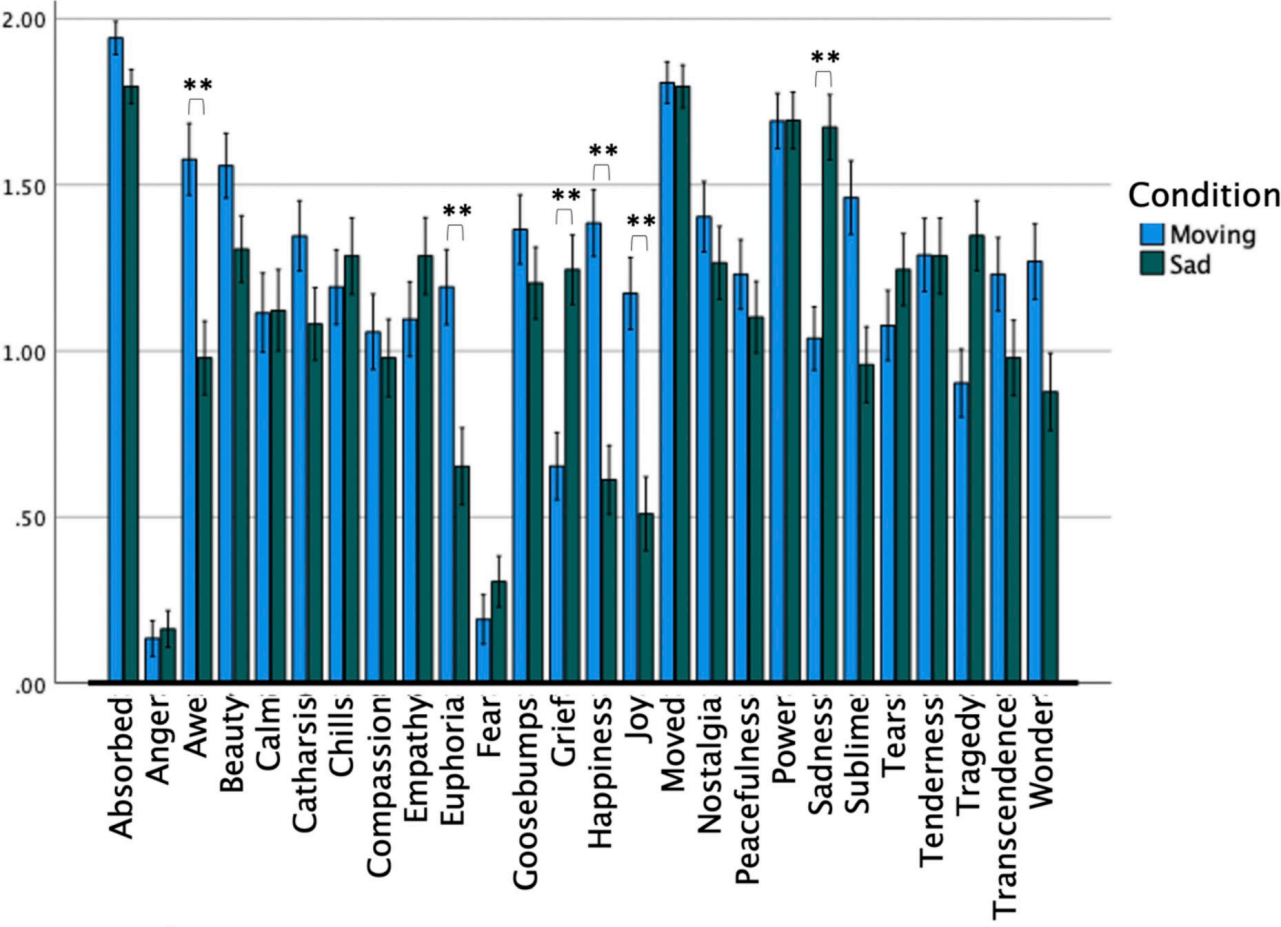
Bar chart of felt affect ratings in response to loved music.

Means for each affect term by condition are summarised in Fig 1. Ratings of the same affect term between conditions were analysed using Bonferroni adjusted independent samples t-tests. Felt sadness was rated higher in the Sad condition, but (non-significantly) higher ratings were given to felt Power, Moved and Absorption ratings in the Sad condition. For the Moved condition the affect term Being moved was rated as the second highest scale (second to Absorption), and the rating was statistically the same as for the rating of Being Moved in the Sad condition. Other differences within and across the two conditions can be observed in Fig 1. Differences for within conditions are not shown because of the large number that were significantly different at p = .05. The highest scoring (with mean rating in at least one condition > 1.5) affect terms were Absorption, Awe, Beauty, Moved, Power, and Sadness.

In these data, a relatively high rating of Being moved can be observed in the Sad condition, and it received a higher rating than the target emotion (Sadness) by M = .122, though non-significantly (p = 1.0), which could be taken to support the action of a mediator, being moved, as responsible for the pleasure generated by the music, despite the accompanying rating of sadness.

### Affects that add to enjoyment

The above results indicate the presence of emotion during the enjoyable music experience. However this does not necessarily confirm that the emotion itself is implicated in the enjoyment of the music. The next step of the study addressed this with an explicit question about the contribution of each affect term to the enjoyment of the music. The 26 Affect terms were presented again this time to be classified as contributing, not contributing, or being irrelevant to the enjoyment of the music. Table 4 lists the counts across each of the three possibilities for each Affect term, by Condition. Chi-Square tests identified whether the Affect words add to enjoyment of the music by chance or not.

**Table 4:** Affect words that add to the enjoyment of the music by condition.

	—		|		+				
Affect term	M	S	M	S	M	S	χ^2^ (M)	χ^2^ (S)	χ^2^ (M&S)
Absorption	4	4	4	4	92	92	83.400*	73.500*	156.85*
Anger	33	32	52	57	15	11	10.423	15.489*	25.515*
A sense of awe	8	13	6	28	87	60	66.269*	16.128*	73.273*
Feeling of beauty	17	4	8	26	75	70	41.346*	31.957*	69.515*
Calm	19	9	19	30	62	62	18.615*	20.213*	37.152*
Chills	19	13	17	21	63	66	21.269*	23.021*	43.818*
Compassion	21	17	15	21	63	62	21.500*	17.149*	38.242*
Empathy	23	11	17	15	60	74	16.423*	35.915*	49.515*
Euphoria	21	11	15	57	63	32	21.500*	15.489*	15.697*
Fear	37	38	54	53	10	9	15.500*	14.596*	30.061*
Sublime	21	19	8	40	71	40	34.885*	4.255	24.182*
Goosebumps	17	15	17	21	65	64	24.038*	19.957*	43.818*
Grief	19	23	35	6	46	70	5.692	30.809*	26.182*
Happiness	23	19	8	43	69	38	32.000*	4.383	20.182*
Joy	21	17	12	49	67	34	27.731*	7.191	16.242*
Being moved	10	2	0	2	90	96	33.923*	82.383*	158.606*
Nostalgia	15	6	15	17	69	77	30.154*	40.383*	69.515*
Peacefulness	25	15	15	30	60	55	16.885*	11.787	26.242*
Powerful feelings	6	6	6	11	88	83	71.115*	52.255*	122.970*
Release/Catharsis	17	6	12	36	71	57	33.731*	18.553*	45.515*
Sadness	10	13	19	4	71	83	34.192*	52.638*	84.061*
Crying	6	19	23	15	71	66	35.808*	22.638*	56.424*
Tenderness	15	15	21	19	63	66	21.500*	22.638*	44.061*
Transcendence	10	11	19	32	71	57	34.192*	15.489*	47.091*
Tragedy	17	30	37	15	46	55	6.731	11.787	13.273*
Wonder	19	19	13	28	67	53	27.269*	8.851	33.152*

Note

M Moving Condition.

S Sad Condition.

**—** Percentage of responses for: ’Emotion felt DOES NOT add to the pleasure/attraction/liking/enjoyment of the piece’.

**|** Percentage of responses for: ’DON’T KNOW / NOT RELEVANT’.

**+** Percentage of responses for: ’Emotion felt ADDS TO THE pleasure/attraction/liking/enjoyment of the piece’.

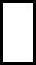
 Thick border around cell indicates category is significantly overrepresented according to Chi-Squared test, df = 2, at p = .05 with Bonferroni adjustment for multiple comparisons. Triplates (of—, **|** and **+**) with no thick border indicates that each of the three categories occur by chance.

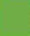
 Darkness of green colour indicates proportionally higher count for ’Emotion felt ADDS TO THE pleasure/attraction/liking/enjoyment of the piece’ (**+**) responses.

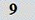
 Green font χ^2^ value indicates statistically significant above-target counts and χ^2^ value > target affect term.

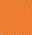
 Darkness of red colour indicates proportionally higher count for ’Emotion felt DOES NOT add to the pleasure/attraction/liking/enjoyment of the piece’ (**—**) OR ’DON’T KNOW / NOT RELEVANT’ (**|**) responses.

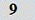
 Red font χ^2^ value indicates that the above-target count for—or **|** is statistically significant.

__Underlined terms are nominally negative in valence.

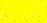
 Yellow highlight is the target affect term in either the Sad or Moved condition.

Significant Chi-square test statistics (at p = .05 with Bonferroni correction) ranged from 15.500 (Fear) to 83.400 (Absorption) for the Moving condition and 14.596 (Fear) to 82.383 (Being moved) for the Sad condition (at p = .05). Chi-Squared tests for Sad and Moving conditions pooled produced statistically significant results for all emotions at p = .05 with Bonferroni correction, ranging from *χ*^2^ = 13.273 (Tragedy) to 158.606 (Being Moved), with second highest *χ*^2^ = 156.85 (for Absorption) and third highest *χ*^2^ = 84.061 (for Sadness).

Self-selected sad music was associated with good likelihood of reporting felt sadness as adding to the pleasure of the experience (83% of response in the Sad condition versus 71% in the Moving condition). The same applies for the affect term rating of Being moved in the Moving condition.

All emotions contributed to the enjoyment of the self-selected music, with the exception of Anger, Fear, Tragedy (both conditions for each, though Tragedy was approaching significance), Grief (Moving condition), Euphoria, Sublime, Happiness, Joy, Peacefulness and Wonder (Sad condition for each). Absorption and Being Moved made the most consistently positive contribution to enjoyment of music, with each being reported as contributing to enjoyment by 90% or more of participants regardless of condition (Table 4).

Fewer nominally negative emotions add to enjoyment in the Moving condition, whereas fewer positive emotions add to enjoyment in the Sad condition. Sadness and crying are emotions with nominally negative connotations, but were reported as adding to the pleasure, regardless of the condition.

### Additional emotions that add to liking

The 26 Affect terms might not have exhaustively covered all the emotions that could be experienced, or enjoyed. Therefore, a final question invited participants to list any other emotions that added to the enjoyment of the music.

Only one expression was reported by different participants more than once—Hopelessness (3 independent mentions, one in the Moving condition). 72 participants indicated that no additional emotions contributed to enjoyment (36 in the Moving condition and 36 in the Sad condition). A higher proportion of participants who did report additional emotions mentioned ones that could be interpreted as negative in the Sad condition compared to the Moving condition, but because of the heterogeneity of the responses, which included some words that were already among the 26 Affect terms, no strong conclusion can be drawn, except that the set of Affect terms was effective in identifying the feelings implicated in pleasurable musical experiences.

### Hypothesis test–Sadness is liked because the music is sad

For the responses to the Sadness removed step, the following scoring was applied to responses: -2 for ‘I would like the piece a LOT LESS’, -1 for ‘I would like the piece a LITTLE LESS’, 1 for ‘I would like the piece a LITTLE MORE’, 2 for ‘I would like the piece a LOT MORE’, and 0 for NO DIFFERENCE. If the Direct effect hypothesis is supported, we would expect liking to reduce when sadness is removed from the experience. The Indirect effect hypothesis, on the other hand, predicts that removal of sadness would not change liking (change of 0) or increase liking. A single sample t-test supported the Direct effect hypothesis, with an overall reduction of .83 (SD = .916) in liking on the scale of -2 to +2 (t(46) = -.6.207, p < .001, Cohen’s-d = .916). For comparison, in the control condition, removal of movingness also led to a reduction in liking (M = -.77, SD = .807, t(51) = -.6872, p < .001, Cohen’s-d = .807). Taken together the data from this step of the study supports the Direct effect hypothesis.

## Discussion

Based on an overall interpretation of the data, the Direct effect hypothesis is supported. In the specific part of the study that tested the hypothesis, the Sadness removed step, participants reported overall significant reduction in pleasure if the felt sadness, and only the felt sadness evoked by the music, were excised. If sadness were not in itself enjoyed, we may have expected participants to attribute non-sad emotions to the enjoyment, or be unable to perform the task. As it turned out, we can confirm that 83% of participants could perform the task and verify that the sadness was specifically enjoyed, suggesting that the phenomenon of interest is empirically demonstrable. To further ascertain if this is a plausible interpretation, the results are interpreted through the alternative, Indirect effect hypothesis, lens by examining whether mediators still play a commensurate or dominant role in the effect.

### Mediation explanation

In the results where affect terms were all rated, a term can be viewed as a mediator if its score or count is statistically equal to or higher than the score or count of the target emotion. Based on this criterion, several steps of the study could be interpreted as supporting the presence of a mediator. In the Open-ended felt emotions step Nostalgia, a prospective mediator of sadness-enjoyment, was spontaneously reported (Table 3). However, Being moved was not, despite previous evidence that Being moved is the stronger candidate of the two [15]. Nostalgia appeared frequently in the Moved condition as well, but in the Moved condition no mediator was expected because the target emotion (being moved) itself already contained an implicitly positive component. Furthermore, Sadness was also frequently reported in the Moved condition, but, again, there is no reason that being moved would require a mediator. The Indirect effect hypothesis does not predict a mediator that is itself negatively valenced. Thus a mediator based explanation for these results is not straight forward.

In the Affects felt step a more credible impact of prospective mediators can be observed. In the Sad condition, Absorbed (rated highest, with M = 1.796), Being moved (rated higher than Sadness by M = .122, though non-significantly [NS], p = 1.0) and Powerful feelings (rated higher than Sadness by M = 0.020, NS p = 1.0) are all rated as high or higher than the target emotion (Sadness). In the Moved condition only Absorbed (M = 1.942) is rated higher than Being moved (by M = .135, NS p = .074). If we set aside the finding for the Moved condition, the mediator-based explanation is supported, triangulating extant evidence that two of these affects (absorbed and moved) are mediators of sadness.

So it is possible to find support for the Indirect-effect hypothesis, and the mediator-based explanation in particular. However, the findings refer to the presence of emotions. There is no assurance that any of the emotions identified are adding to the pleasure, with the exception of the target emotion, since that requirement was made explicit in the procedure.

The Affects that add to liking step addressed the matter. Being moved, Absorption, and Powerful feelings (but not Nostalgia) all had the same or higher counts than the target (Sadness) emotion, indicating that they add to enjoyment in the Sad condition (Table 4). For example, the affect term Being moved was voted as ’adding to pleasure’ by 96% of participants in the Sad condition, compared to the affect term Sadness ’adding to pleasure’ according to 83% of participants. This supports the Indirect effect hypothesis (Table 4).

Here we have the strongest evidence of mediators in explaining enjoyment of sadness, and this aligns with evidence from previous research [as discussed in the introduction, see 17]. But Absorption (adds to pleasure according to 92% of participants) also has a higher count than the target emotion (90%) in the Moved condition. Does that mean that Absorption also mediates Being moved? As pointed out above, that seems unlikely because Being moved already contains a positive aspect, and so should not need a mediator. Using the mediator-based explanation, Absorption adding to enjoyment votes should have (at least) been fewer than the votes for Being moved in the Moving condition (which was not the case). Furthermore, in the Sadness condition, the target emotion itself received statistically significant votes as adding to pleasure, meaning that the alleged mediators may not have served any essential purpose in contributing to the enjoyment. The mediation explanation is only able to partially explain the results. An alternative explanation is proposed by applying the concept of ‘semantic overlap’.

### Semantic overlap explanation

Semantic overlap is a phenomenon concerned with the mental organisation of concepts and word meanings. Words with similar meanings (synonyms) are more linked with one another in a mental space than words with unrelated meanings. This is often characterised in network inspired models of the mind, foundationally proposed by Quillian and the notion of the semantic network [74,75]. Word meanings are organised in a complex yet systematic manner according to network principles, of particular interest here being through similarities in the meaning of words, where expressions that are more similar in meaning appear ‘closer together’ in the mental network. This means that when a word is triggered (e.g., heard or read), the semantically more closely related words are more primed (ready to be raised to conscious attention) in the mental network than less closely related words. Cognitive linguists by and large agree that words are pointers or approximate representations of concepts and experiences stored in memory [76,77]. The implication is that words can be mapped onto points in multidimensional semantic space, with distance between words reflecting (of interest here) degree of conceptual dissimilarity between the words. Considerable effort has been devoted to organising emotions by similarity [e.g., 78–83]. Semantic distance may therefore explain why Being moved frequently appears for sad evoking music (a frequently reported result), and the novel findings identified in the present study.

It is possible to estimate the relative semantic distance between the two words moving and sadness by looking up the terms in a published list of words with quantified point estimates of locations in theoretical semantic space. A large such database was developed by Mohammad [82], where estimates of location in semantic space of some 20,000 English words were produced. The semantic space in that research adopts a conventional representation of the space, particularly relevant for emotions, referred to as ‘VAD’ space. Emotions can be reasonably well expressed in terms of two dimensions, labelled valence (V) and arousal (A), where the former refers to the positive or negative aspect of the word’s meaning (e.g., happy and calm exhibit positive valence, while sad and angry negative) and the degree of activity associated with the word’s meaning (e.g., joyous and furious are high arousal, while calm and sad are low arousal). Some have argued that two dimensions are only partially sufficient for describing the meaning of an emotion [81,84–87], and a frequently proposed third dimension is dominance (D) (where words such as angry and energetic exhibit high dominance, while fear and innocuous are low in dominance), leading to the VAD (Valence, Arousal, Dominance) abbreviation for this three dimensional configuration [other examples: 85, 88, for a review, see 89,90]. Mohammad (82) provided numerical VAD scores for each term scaled to a score between 0 and 1 (negative to positive for valence, low to high for arousal and for dominance) based on human ratings. From these data it is possible to estimate the semantic distance between emotions.

Through calculations using the VAD word list published by Mohammad (82), Moved and Sadness have a semantic distance in VAD space of 0.607 units (numbers closer to 0 indicating greater similarity). With Sadness as the reference, positive emotions appearing in the Affect term list have distances that range from 0.852 for Calm to 1.243 for Joy (all greater than the distance between Sadness and Moving), while negative emotions have scores ranging from 0.469 for Grief (closest negative emotion to Sadness from the Affect terms presented) to 0.768 (Anger), which apart from Anger are all closer to Sadness than Moving is to Sadness. That is, Moving has more semantic overlap with Sadness than does Anger and the positive emotions Joy and Happiness, suggesting semantic overlap as a viable alternative to mediation as to why being moved appears in tandem with sadness. The VAD data also suggest that Moving is semantically more closely related to Sadness than Catharsis, since Catharsis has a distance of 0.633 from Sadness (slightly more distant than Moving). High ratings of Moving for a Sad-evoking context can therefore be explained by semantic overlap. Such an interpretation strengthens the case for supporting the Direct effect hypothesis, because being moved need not be treated as surrogate for sadness.

The Direct effect hypothesis proposes that pleasure is experienced by contextualised re-appraisal or ‘dissociation’ of the Action tendency component of an otherwise negative emotion. The consequent positive experience (enjoyment, pleasure, preference) provides another clue for the remaining Affect terms that were rated the same or higher than the target emotion in each condition. The mediation account fails to explain why Sadness was voted (by 71% of participant) as adding to enjoyment in the Moving condition. The mediator based explanation is also poor at explaining why Absorption was reported as adding to enjoyment, and for doing so in both conditions.

The semantic overlap approach can better explain these results, too. Affect terms such as Absorption and Powerful feelings are affects related to enjoyment when experiencing art. Consider the Absorption in Music scale developed by Sandstrom and Russo [91]. The 34 item scale contains several items related to the pleasure of being engaged with music in different ways [see also 2,18,92,93]. Powerful experiences are reported during special, personal experiences that occur during strong positive aesthetic experiences [94–96, p. xiv]. That is, the task itself, of identifying a loved piece of music, also produces semantic overlap of these terms. Furthermore, in the Sad condition several positive emotions were reported more frequently as having *no relevance* to enjoyment, in comparison to the Moved condition: Euphoria (57% in the Sad condition versus 15% in the Moving condition), Happiness (43% vs 8%) and Joy (49% vs 12%). Mediation struggles to explain why purely positive affect terms are not voted as adding to enjoyment. Semantic overlap, on the other hand, suggests that the activation of sadness is more likely to be associated with other negative emotions, while being moved would be more associated with emotions of both positive and negative valence. In addition to the possibly misleading interpretations of enjoyed-sadness in music research employing a mediator-based approach to explaining the phenomenon, discussed in the Method section, semantic overlap offers an explanation of the results that is superior to the mediator-based explanation.

## Conclusions

This study investigated whether the experience of sadness, evoked by music, can itself be highly enjoyable. A novel method was applied where participants were asked to imagine how enjoyment would be impacted should the felt sadness somehow be removed. The results demonstrated that sadness is directly implicated in the enjoyment of such music, providing support for the ‘Direct effect hypothesis’. This hypothesis states that when sad music is enjoyed, the sadness itself directly contributes to the enjoyment. A theoretical position has been presumed by the hypothesis–that the experience of sadness contains a component that can be dissociated from regular experience of the negative emotion when contemplating music or any aesthetic event. The presence of emotions such as being moved were explained by the concept of semantic overlap, where an emotion concept is not activated as a lexical singular, but rather as the meaning that the emotion encompasses, or that is spread to other related emotions, according to how similar they are (in this case to the concept of sadness). Being moved is sufficiently close in meaning to sadness to allow it to be activated during a sadness evoking music experience, regardless of the extent to which it is enjoyed, meaning that the presence of an emotion such as being moved does not necessarily explain (and is not needed to explain) why felt sadness can be enjoyed. Absorption is another affect that accompanied loved, sadness-inducing music. This, too, was explained by semantic overlap, with the positive component of the sadness activating other, reasonably nearby, positive affects, including Absorption. The state of absorption may also play a causal role in attraction to music [20,97], and so there could well be some feedback loop between absorption and other aspects of the experience, including evoked emotions. Suggestions were made for further research to test whether the semantic overlap account and the Direct effect hypothesis better characterise enjoyment of negative emotion in music than mediators (such as being moved and absorption) that themselves have a positive component, through which enjoyment is indirectly generated.

The results of the present study were enhanced by applying a modified version of research using self-selected stimuli that minimised demand characteristics, while ensuring that the phenomenon of interest was investigated. Methodologically, the study took the critical step of ensuring that the impact of particular affects on enjoyment of the music were investigated, not just their presence. Future research is likely to continue the more popular method of using experimenter-selected stimuli which are then rated along various affect terms. This paper made recommendations on how such research could be more successful at identifying the phenomenon of interest, and in so doing better address the debate on the enjoyment of felt sadness and other felt negative emotions in music.
